# Genetic Evaluation and Pregnancy Outcomes in Foetuses With Overgrowth at a Tertiary Referral Center

**DOI:** 10.1111/jcmm.71162

**Published:** 2026-07-27

**Authors:** Xiaoqing Wu, Qingmei Shen, Xiaorui Xie, Yuqin Chen, Bin Liang, Meiying Wang, Danhua Guo, Na Lin, Liangpu Xu

**Affiliations:** ^1^ Fujian Maternity and Child Health Hospital, College of Clinical Medicine for Obstetrics & Gynecology and Pediatrics Fujian Medical University Fuzhou Fujian China; ^2^ Fujian Provincial Key Laboratory for Prenatal Diagnosis and Birth Defect Fuzhou Fujian China; ^3^ Department of Laboratory Medicine Fujian Medical University Fuzhou Fujian China; ^4^ Key Laboratory of Clinical Laboratory Technology for Precision Medicine (Fujian Medical University) Fujian Province University Fuzhou Fujian China

**Keywords:** biometric parameters, chromosomal abnormalities, chromosomal microarray analysis, fetal overgrowth, karyotyping

## Abstract

Fetal overgrowth is defined as one or more biometric parameters exceeding the 90th–97th percentile or 2 standard deviations above the mean for gestational age. This study aimed to evaluate genetic findings in foetuses diagnosed with sonographic overgrowth. We retrospectively analysed 78 singleton pregnancies that underwent invasive prenatal diagnosis between 2018 and 2024. Fetal overgrowth was defined based on biparietal diameter (BPD), head circumference (HC), and/or abdominal circumference (AC), and cases were categorized into three groups according to the pattern of enlargement: head enlargement (Group A, *n* = 49), abdominal enlargement (Group B, *n* = 12), and combined head and abdominal enlargement (Group C, *n* = 17). All foetuses underwent conventional karyotyping and single nucleotide polymorphism array analysis, and were further classified as isolated or non‐isolated based on the presence of additional ultrasound findings. Chromosomal abnormalities were identified in 2 cases (2.6%) by karyotyping, including mosaic 47,XYY/45,X and 46,X,add(X)(p22)/45,X, while SNP array detected copy number variants involving chromosome 11 in 2 additional cases, one likely pathogenic and one variant of uncertain significance. Notably, all chromosomal abnormalities were observed in the non‐isolated overgrowth group. Overall, 12 cases (15.4%) were classified as isolated and 66 (84.6%) as non‐isolated. Associated ultrasound findings included structural anomalies in 18 cases, non‐structural abnormalities in 12 cases, soft markers in 28 cases, and multiple coexisting abnormalities in 20 cases. Ventriculomegaly was the most common structural anomaly, particularly among foetuses with head enlargement, while polyhydramnios was the most frequent non‐structural finding. Follow‐up data were available for 76 cases, with 8 pregnancies resulting in stillbirth or termination and 68 in live births. Foetuses with combined head and abdominal enlargement had a significantly higher incidence of macrosomia compared with those with isolated head or abdominal enlargement (29.4% vs. 4.3% and 8.3%, respectively; *p* = 0.015). In conclusion, fetal overgrowth is predominantly non‐isolated, and chromosomal abnormalities are mainly observed in this subgroup. Concurrent head and abdominal enlargement is associated with increased risks of structural anomalies and macrosomia. Prenatal genetic testing and macrosomia‐specific management should be considered for head‐abdominal enlargement foetuses.

**Abbreviations**
AC: abdominal circumference
ACMG: American College of Medical Genetics guidelines
ACOG: American College of Obstetricians and Gynaecologists
BPD: biparietal diameter
BWS: Beckwith‐Wiedemann syndrome
ChAS: Chromosome Analysis Suite
CMA: chromosomal microarray analysis
CNV: copy number variation
DECIPHER: Database of Chromosome Imbalance and Phenotype in Humans Using Ensemble Resources
DGV: Database of Genomic Variants
EFW: estimated fetal weight
FGR: Fetal growth restriction
HC: head circumference
ISCN: International System for Human Cytogenomic Nomenclature
LGA: large‐for‐gestational‐age
MRI: magnetic resonance imaging
SNP: single nucleotide polymorphism
TOP: termination of pregnancy
VOUS: variants of unknown significance

## 1 Introduction

Standard fetal biometric parameters, including biparietal diameter (BPD), head circumference (HC), and abdominal circumference (AC), are fundamental components of routine prenatal ultrasound assessment. These measurements are essential for estimating gestational age, evaluating fetal growth patterns, and identifying potential central nervous system abnormalities. Fetal overgrowth is generally defined as one or more biometric parameters exceeding the 90th–97th percentile or more than two standard deviations above the mean for gestational age. Clinically, it may present as large‐for‐gestational‐age (LGA), isolated macrocephaly, or generalized macrosomia.

Macrocephaly, defined as an HC greater than the 98th percentile (> 2 SD above the mean), represents a segmental overgrowth phenotype [1]. Although most cases are familial and benign, macrocephaly may occasionally be associated with intracranial structural abnormalities or underlying genetic syndromes, necessitating careful sonographic evaluation and, in selected cases, further assessment with fetal magnetic resonance imaging (MRI). In contrast, macrosomia is typically defined as a birth weight exceeding 4000 g and occurs in approximately 7%–10% of pregnancies [2, 3]. It is associated with increased risks of obstetric complications, including birth injury, caesarean delivery, and long‐term metabolic consequences in offspring.

Fetal growth regulation is a complex, multifactorial process involving genetic determinants, endocrine and metabolic factors, placental nutrient and oxygen transfer, as well as environmental exposures such as toxins, pollutants, and infections [4, 5, 6]. When fetal overgrowth arises from metabolic imbalance or constitutional factors, such as maternal diabetes or familial growth potential, it is often an isolated finding. In contrast, overgrowth caused by genetic abnormalities is frequently accompanied by additional features, including structural anomalies, dysmorphic characteristics, and neurodevelopmental impairment. Beckwith–Wiedemann syndrome (BWS; MIM #130650) is the most common overgrowth syndrome, with an estimated prevalence of approximately 1 in 10,500 live births, although this figure may be underestimated due to incomplete or atypical phenotypes [7, 8].

In our clinical practice, pregnancies with fetal overgrowth routinely undergo detailed ultrasound surveillance and invasive prenatal genetic testing. In this study, we retrospectively analysed pregnancies complicated by fetal overgrowth between 2018 and 2024, focusing on the diagnostic yield of conventional karyotyping and single nucleotide polymorphism (SNP) array analysis, as well as their correlation with prenatal imaging findings.

## 2 Materials and Methods

### 2.1 Patients and Samples

We conducted a retrospective study on pregnancies complicated by fetal overgrowth between September 2018 and December 2024. The study included 78 singleton pregnancies referred for invasive prenatal diagnosis due to large fetal biometric parameters. The cohort was categorized into three groups based on the pattern of enlargement: Group A (*n* = 49): enlarged head parameters (BPD and/or HC); Group B (*n* = 12): enlarged AC; Group C (*n* = 17): concomitant enlargement of both head and abdomen parameters. The prenatal specimens were sampled via amniocentesis (*n* = 60) or cordocentesis (*n* = 18) for conventional karyotyping and SNP array analysis. Descriptive Characteristics of the study population are summarized in Table 1. Ethical approval was granted by the Institutional Ethics Committee of Fujian Provincial Maternity and Children's Hospital (NO: KLR620), and written informed consent was obtained from all participants. The study was conducted in accordance with the ethical guidelines of the Declaration of Helsinki.

**TABLE 1:** Demographic characters for 78 pregnancies with Enlarged Biometric Parameters.

Characteristic	
Maternal age (years), (range, median, mean ± SD)	23–39, 31, 30.5 ± 3.8
Gestational age at diagnosis(weeks), (range, median, mean ± SD)	18–32, 26, 25.4 ± 3.3
Gestational age at invasive prenatal diagnosis (weeks), (range, median, mean ± SD)	19–34, 30, 28.8 ± 3.7
Specimen	
Amniotic fluid (*n*, %)	60, 76.9
Cord blood (*n*, %)	18, 23.1
Large biometric parameters	
Head (BPD and/or HC) (*n*, %)	49, 62.8
Abdomen (AC) (*n*, %)	12, 15.4
Both head and abdomen (*n*, %)	17, 21.8

Abbreviations: AC, abdominal circumference; BPD, biparietal diameter; HC, head circumference.

### 2.2 Method

#### 2.2.1 Diagnostic Criteria for Fetal Overgrowth

Ultrasound examination was performed by certified sonographers using standard protocols. Gestational age was determined using the best obstetrical estimate, following the American College of Obstetricians and Gynaecologists (ACOG) guidelines. Fetal overgrowth was diagnosed when BPD, HC, and/or AC exceeded 2 SD above the mean for the gestational age based on Hadlock biometric charts. Estimated fetal weight (EFW) > 90th percentile was also recorded but not used as a sole inclusion criterion. Cases were classified as isolated if no other ultrasound anomalies (structural malformations or soft markers) were identified, and as non‐isolated if any additional ultrasound findings were present.

#### 2.2.2 Conventional Karyotyping

Amniotic fluid cells were cultured for 7 days before mitotic arrest with colchicine. Cells were then subjected to hypotonic treatment, fixation, slide preparation, trypsin digestion, and Giemsa staining to produce G‐banded chromosome slides. Lymphocytes from umbilical cord blood were cultured in phytohemagglutinin‐stimulated media for 68–72 h, entering mitosis. Karyotyping was performed at a resolution of 320–500 bands, in accordance with local laboratory protocols. Karyotypes were described according to the International System for Human Cytogenomic Nomenclature (ISCN) 2024 [9].

#### 2.2.3 SNP Array Analysis

Genomic DNA was extracted from uncultured amniotic fluid and fetal cord blood using the QIAamp DNA Blood Mini kit (QIAGEN, Germany). SNP array analysis was performed using the Affymetrix CytoScan 750K array, comprising over 200,000 SNP markers and 550,000 copy number variation (CNV) markers. Raw data were analysed using Chromosome Analysis Suite (ChAS) software version 3.2. Identified CNVs were interpreted by comparison with public databases, including the Database of Genomic Variants (DGV), the Database of Chromosome Imbalance and Phenotype in Humans Using Ensemble Resources (DECIPHER), the International Standards for Cytogenomic Arrays Consortium, and the Online Mendelian Inheritance in Man (OMIM). All variants were classified according to the American College of Medical Genetics (ACMG) guidelines: pathogenic, benign, likely pathogenic, likely benign, and variants of unknown significance (VOUS). In clinical practice, parental testing is generally recommended to clarify the inheritance pattern of identified CNVs, including VOUS. In the present study, VOUS were not considered pathogenic findings and were therefore not included in the calculation of clinically significant detection rates; instead, they were reported separately and interpreted with caution.

#### 2.2.4 Follow Up

All prenatal ultrasound and/or MRI findings were systematically recorded and analyzed. In our clinical practice, fetal brain MRI was recommended primarily for cases with head circumference exceeding the 97th percentile. However, not all eligible patients consented to undergo MRI examination. Therefore, only a subset of foetuses underwent MRI evaluation, resulting in a limited sample size. Due to this selective application and potential selection bias, MRI findings were not included in the statistical analysis but were described descriptively when available. Foetuses with any additional ultrasound abnormalities, including structural anomalies, soft markers, or other non‐structural findings, were classified as non‐isolated cases.

Pregnancy outcome data were obtained from the hospital's electronic medical records or through direct telephone follow‐up with the patients. Outcomes included live birth, termination of pregnancy (TOP), or stillbirth. Stillbirth is defined as intrauterine fetal death at ≥ 20 weeks gestation. Neonatal information, including the mode of delivery and birth weight, was also recorded. Macrosomia was defined as a birth weight ≥ 4000 g.

#### 2.2.5 Statistical Analysis

All statistical analyses were conducted using SPSS software v26.0 (SPSS Inc., Chicago, IL, USA). Categorical comparisons were performed using the chi‐square test or Fisher's exact test, as appropriate. A two‐tailed *p* < 0.05 was considered statistically significant.

## 3 Results

### 3.1 Genetic Findings

**FIGURE 1:**
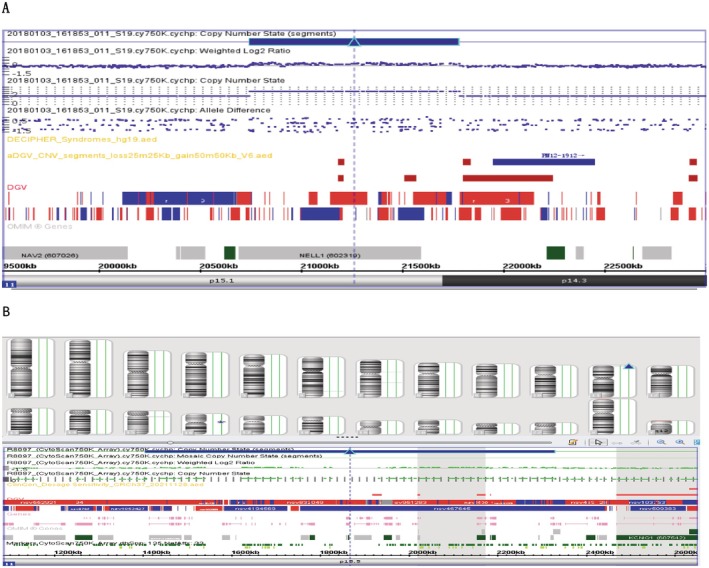
The results of SNP array analysis for the 2 cases of VOUS. (A) a 924 kb duplication at the region of 11p15.5; (B) a 1.0 Mb duplication at the region of 11p15.1‐p14.3.

Chromosomal abnormalities were identified in 2 of 78 foetuses (2.6%) by both conventional karyotyping and SNP array analysis. One case (Case 1) exhibited mosaicism for 47,XYY/45,X, and another (Case 2) showed mosaicism for 46,X,add(X)(p22)/45,X. The pregnancy in Case 1 was electively terminated, whereas Case 2 resulted in stillbirth. SNP array analysis revealed two additional CNVs involving chromosome 11. Case 3 carried a 924 kb duplication at 11p15.5 (Figure 1A), defined as arr[GRCh37]11p15.5 (1,403,188–2,327,070) × 3, which was classified as likely pathogenic. The pregnancy was subsequently terminated. Case 4 harboured a de novo 1.0 Mb duplication at 11p15.1p14.3 (Figure 1B), defined as arr[GRCh37]11p15.1p14.3 (20,745,930–21,780,075) × 3, classified as a VOUS. Despite the presence of fetal overgrowth and multiple congenital anomalies, this pregnancy resulted in a live birth. The infant was delivered vaginally with a birth weight of 4400 g and demonstrated normal growth and neurodevelopment at a 4‐year follow‐up. Parental testing to determine the inheritance of the two CNVs was declined in both cases. The detection rates of chromosomal abnormalities differed among the three biometric groups but did not show statistical significance: 2.0% (1/49) in Group A, 7.7% (1/13) in Group B, and 10.5% (2/19) in Group C (*p* > 0.05). Details of the four cases with chromosomal abnormalities are summarized in Table 2.

**TABLE 2:** Genetic abnormalities and ultrasound details in pregnancies with large biometric parameters.

Case number	Ultrasound findings	Karyotyping results	SNP array analysis				Pathogenicity classification	Pregnancy outcomes
				Size	Chromosomes or key OMIM genes	Inheritance		
1	Enlarged BPD, EIF, mild tricuspid regurgitation, Bilateral renal pelvis separation	47,XYY [27]/45,X [21]	arr(X)x1, (Y)x1 ~ 2	~59 Mb (whole Y chromosome)	Mosaic Y chromosome gain	dn	Pathogenic	Stillbirth
2	Enlarged BPD and AC, short FL	46,X,add(X)(p22)[69]/45,X [31]	arr[GRCh37] Xp22.33p21.3(168,551–26,023,162)x1~2, Xp21.3q28(26,031,561‐155,233,098)x1~2	25.8 Mb, 129.2 Mb	Mosaic X chromosome loss	dn	Pathogenic	TOP
3	Enlarged AC, hepatomegaly, large kidney, echogenic bowel, polyhydramnios	Normal	arr[GRCh37] 11p15.5(1,403,188‐2,327,070)x3	924 kb	H19, IGF2	Unknown	VOUS	TOP
4	Enlarged BPD and AC, VSD, right hydronephrosis, left ventriculomegaly, cerebellomedullary cisterna magna, ependymal cyst, interpeduncular cyst	Normal	arr[GRCh37] 11p15.1p14.3(20,745,930‐21,780,075)x3	1.0 Mb	NELL1	Unknown	Likely Pathogenic	Live birth, natural delivery

Abbreviations: AC, abdominal circumference; BPD, biparietal diameter; dn, de novo; FL, femur length; HC, head circumference; TOP, termination of pregnancy; VSD, ventricular septal defect.

### 3.2 Associated Ultrasound Abnormalities

Among the 78 cases, 12 (15.4%) were classified as isolated overgrowth, and 66 foetuses (84.6%) were categorized as non‐isolated overgrowth based on the presence of additional ultrasound abnormalities. Associated ultrasound findings included structural anomalies in 18 cases, non‐structural abnormalities in 12 cases, soft markers in 28 cases, and multiple coexisting abnormalities in 8 cases. A detailed summary of associated structural malformations in each group is provided in Table 3. The prevalence of structural anomalies differed among groups: 20.4% (10/49) in Group A, 33.3% (4/12) in Group B, and 11.8% (2/17) in Group C. Ventriculomegaly was the most common structural anomaly, observed in 8 foetuses (10.2%) and occurred predominantly in foetuses with head enlargement. Polyhydramnios was the most frequent non‐structural abnormality, occurring in 16 of 78 cases (20.5%). Among these, three cases (18.8%) were complicated by gestational diabetes mellitus.

**TABLE 3:** Detailed structural malformations in foetuses with large biometric parameters.

Groups	Accompanied malformations	*n*
Group A		11
	Ventriculomegaly^a^	6, 54.5%
	Absent septum pellucidum	2, 18.2%
	Left renal atrophy	1, 9.1%
	Talipes	1, 9.1%
Group B		4
	Hydronephrosis	1, 25.0%
	Enlarged posterior fossa cistern and hypoplasia of the cerebellar vermis	1, 25.0%
	Hepatomegaly and bilateral renomegaly	1, 25.0%
	Enlarged septum pellucidum	1, 25.0%
Group C		9
	Ventriculomegaly^b^	2, 22.2%
	Ventricular septal defect	2, 22.2%
	Short femur	1, 11.1%
	Left renal atrophy	1, 11.1%
	Hydronephrosis	1, 11.1%
	Bilateral renomegaly	1, 11.1%
	Multiple malformation: ventricular septal defect, right hydronephrosis, left ventriculomegaly	1, 11.1%

*Note:* Group A: large BPD and/or HC; Group B: large AC; Group C: both head and abdomen parameters were large.

^a^ Three cases were classified as mild and three as moderate.

^b^ One case was classified as mild and the other one as moderate.

### 3.3 Pregnancy Outcomes

Follow‐up information was available for 76 of 78 (97.4%) cases, with two cases lost to follow‐up. Among the 76 cases with known outcomes, there were 5 terminations of pregnancy due to chromosomal abnormalities or associated structural malformations, 3 stillbirths, and 68 live births. Of the 68 liveborn infants, 35 (51.2%) were delivered by caesarean section and 33 (48.5%) were delivered vaginally. The rate of caesarean delivery varied across groups: 26 out of 45 cases (57.8%) in Group A, 5 out of 10 (50.0%) in Group B, and 4 out of 13 (30.7%) in Group C. These differences were not statistically significant (*p* > 0.05). Neonatal birth weights varied among groups, with mean birth weights of 3565 ± 359 g in Group A, 3700 ± 271 g in Group B, and 3741 ± 505 g in Group C. Macrosomia (birth weight ≥ 4000 g) was observed in 2 of 47 cases (4.3%) in Group A, 1 of 12 (8.3%) in Group B, and 5 of 17 (29.4%) in Group C (*p* = 0.003 for Group C vs. others). A summary of pregnancy outcomes by group is provided in Table 4.

**TABLE 4:** The pregnancy outcomes of 76 foetuses with large biometric parameters.

	Head (BPD and/or HC)	Abdomen (AC)	Both head and abdomen
TOP	1, 2.1%	2, 16.7%	2, 11.8%
Stillbirth	1, 2.1%	0, 0.0%	2, 11.8%
Livebirth	45, 95.7%	10, 83.3%	13, 76.4%
Weight < 4000 g	43, 91.5%	9, 75.0%	8, 47.1%
Weight ≥ 4000 g	2, 4.3%	1, 8.3%	5, 29.4%
Total	47	12	17

Abbreviations: AC, Abdominal Circumference; BPD, Biparietal Diameter; HC, Head Circumference; TOP, Termination of Pregnancy.

## 4 Discussion

Limited publications are available regarding the prenatal evaluation of fetal overgrowth defined by large biometric parameters via ultrasound. In this retrospective study of 78 pregnancies, we comprehensively assessed the genetic findings, associated ultrasound phenotypes, and perinatal outcomes. The key findings include a low overall detection rate of clinically significant chromosomal abnormalities, a high proportion of non‐isolated overgrowth accompanied by additional sonographic findings, and generally favorable neonatal outcomes among live births.

To the best of our knowledge, certain genetic syndromes associated with fetal overgrowth have been reported [10, 11, 12, 13], while systematic investigations on the relationship between fetal overgrowth and chromosomal abnormalities remain limited. In our study, chromosomal aberrations were identified in four non‐isolated cases, including two cases of sex chromosome mosaicism and two CNVs, resulting in an overall detection rate of 2.6%. Case 1 presented with an atypical Turner karyotype. In addition to large head parameters, the fetus also exhibited short femur length, which is consistent with Turner‐associated features. Case 2 showed a karyotype of 47,XYY/45,X mosaicism, for which prenatal phenotypic data are limited. The fetus displayed concurrent enlargement of both head and abdominal measurements, suggesting that carrying an excess of the Y‐bearing cell line may have contributed to an overgrowth phenotype. Case 3 involved a 924 kb duplication at 11p15.5, encompassing 20 OMIM‐listed genes, notably the imprinted genes H19 (103280) and IGF2 (147470). This region includes the imprinting control region IC1, where a paternally inherited duplication may disrupt normal methylation patterns [14]. Hypermethylation of this region has been associated with Beckwith‐Wiedemann syndrome (BWS) [14, 15], a paediatric overgrowth disorder characterized by macrosomia, macroglossia, visceromegaly, omphalocele, earlobe creases or pits, and variable degrees of intellectual development, sometimes normal. However, as parental origin testing and methylation‐specific multiplex ligation‐dependent probe amplification were not performed, the pathogenic mechanism and its relationship to BWS cannot be confirmed. Although the observed fetal phenotypes are suggestive of BWS, this potential association should be interpreted with caution. The pregnancy was ultimately terminated. Case 4, from Group C, exhibited a 1.0 Mb duplication at the region of 11p15.1‐p14.3, encompassing the OMIM gene of NELL1 (602319). While NELL1 has been reported to be involved in bone development [16], the clinical significance of this duplication remains unclear. Although the fetus presented with multiple structural anomalies, the pregnancy resulted in the delivery of a macrosomia, and no abnormal phenotype was observed during a 4‐year postnatal follow‐up. Overall, our findings indicate that in the absence of additional ultrasound anomalies, the likelihood of major chromosomal abnormalities in foetuses presenting primarily with overgrowth is very low.

Although the detection rates of chromosomal abnormalities appeared to increase from Group A to Group C, the differences were not statistically significant. This may be partly explained by the limited sample size in each subgroup.

Most foetuses in this cohort presented with non‐isolated overgrowth, underscoring the importance of systematic ultrasound evaluation once excessive fetal growth is identified. We observed a notable association between large head parameters and ventriculomegaly. In Group A, over half (54.5%) of the accompanied structural anomalies involved ventriculomegaly. Consistent with previous prospective data showing that isolated mild ventriculomegaly could be related to other larger fetal biometric measurements and does not necessarily mean a pathological condition [17]. In this study, some accompanying findings, particularly soft markers, may be incidental and not causally related to fetal overgrowth. Notably, all but one of these cases in our study had favorable postnatal outcomes.

Polyhydramnios affected 20.5% of cases, a rate substantially higher than the 1%–2% observed in the general obstetric population [18]. Previous studies have reported a higher prevalence of LGA neonates in pregnancies complicated by polyhydramnios compared to controls [19, 20, 21]. However, the study by Sarah Crimmins et al. did not find a significant association between accelerated fetal growth and polyhydramnios [22]. Given that maternal diabetes mellitus is a well‐established contributor to both fetal overgrowth [23, 24] and polyhydramnios [22, 25, 26], the coexistence of these findings often warrants maternal metabolic evaluation [27]. In our study, gestational diabetes mellitus was identified in 3 of the 16 cases (18.8%) with concurrent fetal overgrowth and polyhydramnios, closely mirroring the incidence of 15.0% reported in previous literature [25]. All of the three cases were diagnosed based on elevated glucose levels during routine screening at 18–24 weeks of gestation, with no prior history of diabetes. These findings suggest that gestational diabetes may contribute to overgrowth and polyhydramnios in a subset of pregnancies. However, detailed data on maternal glycemic control were not systematically available. In addition, no evidence of other common causes of polyhydramnios, such as fetal swallowing dysfunction or major structural anomalies, was identified in these cases. Therefore, the underlying aetiology of polyhydramnios in this cohort remains unclear, and its contribution to fetal overgrowth should be interpreted with caution.

Fetal overgrowth is associated with worse perinatal outcomes and may carry a higher risk of stillbirth compared with appropriately grown foetuses [3, 28, 29]. In our study, the overall rate of stillbirth was 3.9%, with a higher incidence observed in foetuses exhibiting both enlarged head parameters and AC compared to the other two groups. Mid‐trimester sonographic parameters provide valuable predictive information for LGA infants, or even macrosomia at birth [30]. This condition, in turn, increases the risk of prolonged labor, severe perineal lacerations, and caesarean delivery [31, 32]. As shown in the present study, among the 68 live births, 35 (51.2%) were delivered via caesarean section and 33 (48.5%) via vaginal delivery. Notably, macrosomia was more commonly observed in foetuses with large sonographic parameters both on the head and AC, with a prevalence of 29.4%, significantly higher than in the other two groups and 0.5%–14.9% reported in developing countries [33].

This study has several limitations. First, as a retrospective single‐center analysis, there may be selection bias. Second, the sample size of certain subgroups, particularly Group B and Group C, was relatively small, which may limit the statistical power to detect subtle differences. In addition, long‐term follow‐up data were not systematically available, especially for cases with head enlargement, which limits the assessment of neurodevelopmental outcomes. Furthermore, prenatal imaging has inherent limitations in distinguishing familial macrocephaly from pathological overgrowth, particularly in the absence of detailed family history or postnatal evaluation. Future studies would benefit from more structured follow‐up, with developmental evaluations performed at defined time points (such as between 2 and 5 years of age), to allow a more reliable assessment of prognosis.

## 5 Conclusions

Our findings suggest that fetal overgrowth is frequently accompanied by additional sonographic abnormalities. The risk of chromosomal abnormalities is low in isolated overgrowth but may increase when additional anomalies are present, underscoring the need for further genetic evaluation. Moreover, given the increased risk of macrosomia, careful obstetric planning and individualized perinatal management are essential to optimize pregnancy outcomes. However, given the retrospective design and limited sample size, these findings should be interpreted with caution. Larger, prospective studies are needed to further validate these observations.

## Author Contributions

**Meiying Wang:** investigation. **Qingmei Shen:** writing – original draft, resources. **Xiaoqing Wu:** conceptualization, methodology. **Liangpu Xu:** conceptualization, supervision, project administration. **Bin Liang:** formal analysis. **Na Lin:** supervision. **Xiaorui Xie:** investigation, validation. **Yuqin Chen:** data curation. **Danhua Guo:** data curation, visualization.

## Funding

The study was supported by the Joint Funds for the innovation of science and Technology, Fujian province (grant no. 2025Y9613); Fujian Provincial Health Technology Project (grant no. 2025GGB034).

## Ethics Statement

The present study was approved by the Protection of Human Ethics Committee of Fujian Maternity and Child Health Hospital.

## Consent

Written informed consent was obtained from each pregnant woman.

## Conflicts of Interest

The authors declare no conflicts of interest.

## Data Availability

The datasets used and/or analysed during the current study are available from the corresponding author on reasonable request.
